# Apoptosis mechanisms induced by 15d-PMJ_2_ in HCT116 colon cancer cells: insights into CHOP10/TRB3/Akt signaling

**DOI:** 10.3389/fphar.2023.1283677

**Published:** 2023-11-02

**Authors:** Hussam Albassam, Daniel A. Ladin, Ahmed Elhassanny, Colin Burns, Rukiyah Van Dross-Anderson

**Affiliations:** ^1^ Department of Pharmacology and Toxicology, Brody School of Medicine, East Carolina University, Greenville, NC, United States; ^2^ Department of Chemistry, East Carolina University, Greenville, NC, United States; ^3^ Center for Health Disparities, East Carolina University, Greenville, NC, United States

**Keywords:** Akt, apoptosis, cancer, CHOP10, drug development, endoplasmic reticulum stress, prostaglandin J2, TRB3

## Abstract

Agents that stimulate the endoplasmic reticulum (ER) stress pathway are being exploited pharmacologically to induce cancer cell death. Cytotoxic ER stress is typically regulated by the transcription factor, C/EBP homologous protein 10 (CHOP10). Products of CHOP10 transcription include the pro-apoptotic proteins: ER oxidoreductase 1α (ERO1α), death receptor-5 (DR5), and tribbles-related protein 3 (TRB3). Our previous findings showed cell death induced by 15-deoxy- Δ12,14 prostamide J_2_ (15d-PMJ_2_) occurred in an ER stress-dependent manner. However, the pathway by which 15d-PMJ_2_ regulates ER stress-mediated death downstream of CHOP10 has not been identified. Our results demonstrate 5 µM 15d-PMJ_2_ increased CHOP10 expression and apoptosis in HCT116 colon cancer cells. In cells treated with pharmacological inhibitors of ER stress, 15d-PMJ_2_-induced apoptosis was reliant upon the ER stress pathway. To investigate the role of CHOP10 and its transcriptional products in apoptosis, genetic deletion of CHOP10 (CHOP10-KO) was performed using the CRISPR/Cas9 system. The apoptotic action of 15d-PMJ_2_ was blunted in cells lacking CHOP10 expression. The deletion of CHOP10 reduced the expression of DR5, ERO1α, and TRB3 although only the expression of TRB3 was significantly reduced. Therefore, we overexpressed TRB3 in CHOP10-KO cells and observed that the activation of Akt was inhibited and 15d-PMJ2-induced apoptosis was restored. Thus, a mechanism of apoptosis elicited by 15d-PMJ_2_ includes the stimulation of CHOP10/TRB3/Akt inhibition. Given the important role these signaling molecules play in cancer cell fate, 15d-PMJ_2_ may be an effective inducer of apoptosis in cancer cells.

## Introduction

Cancer is the second most common cause of death in the United States (Siegel et al., 2023). The use of cytotoxic, targeted, and immunostimulatory agents has improved therapeutic outcomes for cancer. In spite of this, novel agents are needed to provide cancer patients with more treatment options and to increase survival rates.

Numerous preclinical and clinical studies have determined that agents targeting the endoplasmic reticulum (ER) stress pathway inhibit the growth and survival of cancer cells (Ladin et al., 2017; Prabhu et al., 2020; Chang et al., 2021; Tian et al., 2021; Botrus et al., 2022). The ER plays an essential role in folding and post-translational modification of secretory and membrane proteins. Disturbances in ER homeostasis compromise protein folding and initiate ER stress (Boyce and Yuan, 2006; Yadav et al., 2014). To alleviate ER stress, the highly conserved stress proteins, inositol-requiring enzyme 1 (IRE1), protein kinase R (PKR)-like endoplasmic reticulum kinase (PERK) and activating transcription factor 6 (ATF6) are activated. Stimulation of these ER sensors prevents protein synthesis, enhances ER chaperone activity, and promotes degradation of misfolded or unfolded proteins to restore ER homeostasis. However, when homeostasis cannot be restored due to ER stress overload, apoptosis is initiated (Boyce and Yuan, 2006; Fu et al., 2021). The apoptotic ER stress pathway is primarily regulated by ER stress sensor-induced expression of CCAAT/enhancer-binding protein (C/EBP) homologous protein 10 (CHOP10) (Li et al., 2014; Yang et al., 2017; Hu et al., 2019). The transcription factor, CHOP10, increases the synthesis of genes that promote cell death including ER oxidoreductase 1α (ERO1α), death receptor-5 (DR5), and tribbles-related protein 3 (TRB3) (Li et al., 2014). ERO1α is an ER luminal molecule that facilitates protein oxidation, a process that can produce cytotoxic levels of reactive oxygen species (Marciniak et al., 2004; Chen et al., 2015; Ramming et al., 2015). DR5, a member of the tumor necrosis factor (TNF) receptor family, induces apoptosis upon engagement of TNF-related apoptosis-inducing ligand (TRAIL) (MacFarlane et al., 1997; Yamaguchi and Wang, 2004). TRB3 is an intracellular pseudokinase that was identified as a protein inhibitor of the anti-apoptotic molecule, protein kinase B (PKB)/Akt (Ohoka et al., 2005; Li et al., 2017).

ER stress-dependent death is induced by cannabinoids in different cancer cell types (Salazar et al., 2009; Almada et al., 2017). The cannabinoid chemical class is composed of phytocannabinoids (plant-derived), synthetic cannabinoids, and endocannabinoids (endogenously synthesized cannabinoids), all of which bind to and initiate many of their effects through cell surface cannabinoid receptors (CBR) (Soderstrom et al., 2017; Lu and Mackie, 2021). Cannabinoids and CBRs are components of the endocannabinoid system (ECS) which also includes the proteins involved in cannabinoid synthesis, degradation, and uptake. The ECS is being investigated to uncover its role in physiological processes including cardiovascular and central nervous system homeostasis (Soderstrom et al., 2017; Sierra et al., 2018; Zou and Kumar, 2018). Other research has examined whether components of the ECS can serve as drug targets or pharmacological agents that modulate conditions including pain, inflammation, and cancer (Nagarkatti et al., 2009; Rahn and Hohmann, 2009; Ladin et al., 2016). Significant anti-tumor activity has been observed in cancer cells exposed to phytocannabinoids, synthetic cannabinoids, and endocannabinoids (Ladin et al., 2016; Hinz and Ramer, 2022). Arachidonoyl-ethanolamide (AEA) is an endocannabinoid that causes death of neoplastic cells including breast, endometrial, and skin cancer (De Petrocellis et al., 1998; Soliman et al., 2016; Fonseca et al., 2018). It has been determined that AEA is metabolized by the enzyme, cyclooxygenase-2 (COX-2), to the prostaglandin-ethanolamides, PGE_2_-EA, PGD_2_-EA and PGF_2_alpha-EA (Yu et al., 1997; Kozak et al., 2002). Our group identified a novel molecule produced from the metabolism of AEA by COX-2 named 15-deoxy-Δ^12,14^ prostamide J_2_ (15d-PMJ_2_) (Soliman et al., 2016; Ladin et al., 2017). 15d-PMJ_2_ activates apoptotic ER stress and ER stress-dependent exposure of damage associated molecular patterns (DAMPs) (Ladin et al., 2022; 2017; Elhassanny et al., 2020). The induction of ER stress by 15d-PMJ_2_ is primarily mediated by PERK signaling which causes the release of ER-resident Ca^2+^ and Ca^2+^ overload in the mitochondria leading to ER-stress dependent apoptosis (Ladin et al., 2022). 15d-PMJ_2_-induced PERK activation also increases the expression of pro-apoptotic CHOP10 (Ladin et al., 2017). However, the molecular mechanisms by which CHOP10 stimulates apoptosis in 15d-PMJ_2_ treated cells are unclear. The current work aims to identify signaling pathways downstream of CHOP10 that are responsible for the apoptotic effect of 15d-PMJ_2_. By identifying signaling pathways of 15d-PMJ_2_-induced cell death, we will obtain a better understanding of its mechanism of action. This information can be used to increase the probability of achieving positive outcomes in future clinical studies (Editorial, 2010; Lin et al., 2019; Jang et al., 2021).

## Materials and methods

### Antibodies and reagents

Thapsigargin was purchased from AdipoGen (San Diego, CA), while tert-butyl hydroperoxide (tBHP) was obtained from Sigma-Aldrich (St. Louis, MO). Antibodies directed toward P-PERK, total-PERK, P-eIF2α, total-eIF2α, P-Akt, and total-Akt were obtained from Cell Signaling Technologies (Beverley, MA). Cleaved caspase-8 and anti-mouse HRP antibodies were acquired from Invitrogen (Grand Island, NY). Anti-CHOP10 and anti-rabbit HRP antibodies were procured from Santa Cruz Biotechnology (Santa Cruz, CA). Anti-GAPDH was obtained from Millipore (Billerica, MA) and anti-ERO1α was purchased from Novus Biologicals (Littleton, CO).

### Cell culture

HCT116 human colon cancer cells were purchased from the American Type Culture Collection (ATCC, Manassas, VA). HCT116 cells were maintained in McCoy’s medium [containing 10% of heat-inactivated fetal bovine serum (FBS), penicillin (100 mg/mL), and streptomycin (100 mg/mL)] as recommended by the manufacturer (Sigma Aldrich, St. Louis, MO).

### Apoptosis detection

Caspase 3/7 activity was detected in cultured cells plated in white-walled 96-well plates and incubated for 48 h. Culture medium containing the appropriate concentrations of 15d-PMJ_2_ was added to the cells for 16 h, the time point of peak caspase 3/7 activity. Caspase-Glo 3/7 reagent (Promega, Madison, WI) was added to each well as directed by the manufacturer and luminescence was detected using the Infinite^®^ M200 PRO multimode reader (Tecan, Switzerland). The percent from untreated was calculated for each well using the formula, [(sample–untreated) ÷ untreated] x 100 and then the average value of triplicate wells was determined. The data from three independent experiments were analyzed.

Apoptotic cells were also detected using the Apoptotic, Necrotic, and Healthy Cells Quantification Kit (Biotium, Hayward, CA). Briefly, HCT116 cells were treated with 15d-PMJ_2_ for 16 h, the time point of maximal Annexin V signal. The cells were detached from the culture dishes, washed twice with phosphate buffer saline (PBS), resuspended in Annexin Binding Buffer containing Annexin V-FITC and ethidium homodimer (EtBr), and then incubated in the dark for 15 min. The samples were diluted with four volumes of Annexin Binding Buffer and analyzed using an Accuri C6 flow cytometer (BD Accuri Cytometer, Ann Arbor, MI) at an excitation wavelength of 488 and 533 ± 30 nm and emission of 565 ± 30 nm.

### Western blot analysis

Cells were plated in 100 mm tissue culture dishes, incubated for 48 h, and agents were added to serum-free culture medium as indicated in the text. The cells were washed twice with ice-cold PBS and 100 μL of triton lysis buffer (containing protease and phosphatase inhibitors) was added to each dish. Protein concentrations were measured using BCA reagents (Thermo Fisher Scientific, Waltham, MA). Equal concentrations of each sample were loaded onto SDS-PAGE gels and protein bands were transferred to polyvinylidene difluoride (PVDF) membranes (BioRad, Hercules, CA) using semi-dry transfer cells (TRANS-BLOTSD; Bio-Rad Laboratories, Hercules, CA). The membranes were incubated at room temperature in blocking buffer (5% non-fat dry milk) for 1 h. Next, the membranes were incubated overnight with blocking buffer containing anti-p-PERK (1:1000), total-PERK (1:1000), anti-p-eIF2α (1:1000), total-eIF2α (1:1000), anti-CHOP10 (1:200), anti-ERO1α (1:500), anti-TRB3 (1:500), anti-DR5 (1:500), anti-pAkt (1:1000), total-Akt (1:1000), or anti-GAPDH (1:10000). The PVDF membranes were then incubated with the appropriate secondary antibody for 1 h. Protein bands were visualized using the enhanced chemiluminescence detection system (Thermo Fisher Scientific, Waltham, MA) and protein signals were detected by utilizing a ChemiDoc imaging system (Bio-Rad Laboratories, Hercules, CA). The protein expression levels were quantified by optical densitometry using ImageJ Software.

### DNA transfection

CRISPR/Cas9 is a genetic modification system that has been adapted from bacteria (Barrangou et al., 2007). In this system, the Cas9 nuclease is directed by a single guide RNA (sgRNA) to its target DNA sequence where a double-stranded structure is created that becomes cleaved by Cas9 nuclease (Jinek et al., 2012). CHOP10 knockout HCT116 cells were generated using the CRISPR/CAS9 system as directed by the manufacturer (Santa Cruz Biotechnology, Santa Cruz, CA). Briefly, in a 6-well tissue culture dish, 2 × 10^5^ cells were seeded in 3 mL of antibiotic-free standard growth medium per well, 24 h prior to transfection. Cells were grown to a 50% confluency. Plasmid DNA/UltraCruz^®^ Transfection Reagent Complex was added to each well in a dropwise fashion and the cells were then incubated for 72 h. After incubation, successful transfection of CRISPR/Cas9 Plasmid was visually confirmed by detection of the green fluorescent protein (GFP) via fluorescent microscopy. Transfected cells were selected by incubation with puromycin (0.8 μg/mL), and different colonies were isolated to measure CHOP10 protein expression. Cell colonies that demonstrate complete knockout of CHOP10 expression (HCT116 CHOP10-KO) were utilized in subsequent experiments.

For CHOP10 overexpression, HCT116 CHOP10-KO cells were cultured in the appropriate plates and/or culture slides and transfected with human CHOP10 cDNA in the pCMV3-C-OFPSpark vector or pCMV3-C-OFPSpark with no cDNA insert (empty vector) (Sino Biological, Beijing, China) using UltraCruz transfection reagent (Santa Cruz Biotechnology, Santa Cruz, CA).

### Immunocytochemistry

DNA transfected cells were treated as indicated and fixed with 2% paraformaldehyde in PBS. The cells were then incubated with permeabilization buffer (0.1% Triton X-100 in PBS) for 10 min and incubated with blocking buffer (1X PBS +3% FBS +0.5% Tween20) for 1 h. The blocked cells were then incubated with the indicated primary antibodies and the appropriate immunofluorescence-tagged secondary antibodies. Images were acquired and analyzed by confocal laser microscopy (ZEISS LSM 700 confocal microscope system). Fluorescence intensity was quantified by utilizing ImageJ software.

### Reactive oxygen species detection

Reactive oxidative species (ROS) in cultured cells were measured using the chloromethyl-2′,7′dichlorodihydrofluorescein diacetate (CM-H_2_DCFDA) probe (Life technologies, Grand Island, NY). Cells were loaded with 5 μM CM-H_2_DCFDA in phenol red-free, serum-free medium for 30 min and then treated for 8 h. The cells were trypsinized, reconstituted in serum containing, phenol red-free medium and DCF fluorescence was measured using an Accuri C6 flow cytometer (BD Accuri Cytometers, Ann Arbor, MI) at an excitation wavelength of 488 nm and an emission of 533 ± 30 nm.

### Statistical analyses

All data are representative of at least three independent experiments. Data are presented as the mean ± standard error of the mean (SEM). One- or two-way Analysis of Variance (ANOVA) followed by Tukey’s or Bonferroni’s *post hoc* analysis was carried out using GraphPad Prism.

## Results

### 15d-PMJ_2_ causes ER stress-dependent apoptosis

**FIGURE 1:**
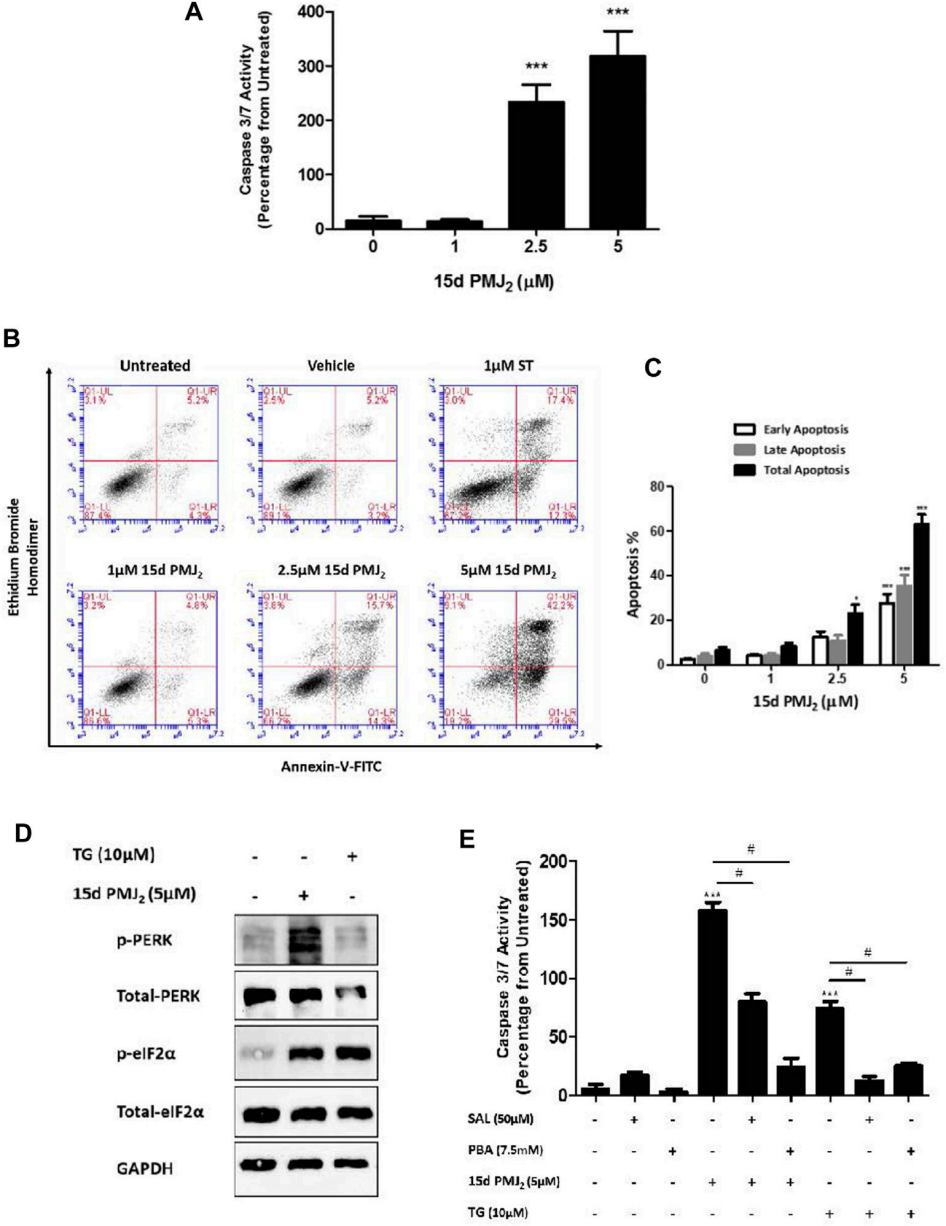
15d-PMJ_2_ causes ER stress-mediated apoptosis in HCT116 cells. **(A)** HCT116 cells were treated with 1 μM, 2.5 µM, and 5 µM 15d-PMJ_2_ or vehicle (culture medium containing ≤0.1% DMSO) for 16 h. Apoptosis was detected by conducting caspase 3/7 activity assays. **(B)** HCT116 cells were untreated or treated with 1 µM 15d-PMJ_2_, 2.5 µM 15d-PMJ_2_, 5 µM 15d-PMJ_2_, 1 µM staurosporine (ST; positive control), or vehicle for 16 h. Flow cytometric analysis was performed to detect Annexin V and ethidium bromide homodimer (EtBr) staining. **(C)** Quantification of flow cytometric analysis data. Histogram shows early apoptosis (Annexin V^+^ EtBr-), late apoptosis (Annexin V^+^ EtBr^+^), and total (early and late) apoptosis in three independent experiments. **(D)** HCT116 cells were treated with 5 µM 15d-PMJ_2_, 10 µM thapsigargin (TG), or vehicle for 4 h. The levels of phosphorylated and total PERK (MW: 125 kDa) and eIF2α (MW: 36 kDa) were determined by conducting western blot analysis with GAPDH (MW: 36 kDa) detected as a loading control. **(E)** HCT116 cells were pre-treated with 50 µM salubrinal (SAL) or 7.5 µM PBA for 30 min. The cells were then treated with 5 µM 15d-PMJ_2_ or 10 µM thapsigargin (TG), and caspase 3/7 activity was measured. The data were analyzed using one-way ANOVA followed by Tukey’s multiple comparison test and are presented as the mean ± SEM of three independent experiments performed in triplicate. *p < 0.05 and ***p < 0.001 when comparing samples to vehicle-treated cells. #*p < 0.05*, when comparing samples to 15d-PMJ_2_-treated cells.

Previous data from our group demonstrated AEA and its metabolic product, 15d-PMJ_2_, caused apoptosis in melanoma and non-melanoma skin cancer cells through ER stress-stimulated CHOP10 expression (Soliman et al., 2016; Ladin et al., 2017). The current study sought to identify signaling pathways downstream of CHOP10 that initiate this cytotoxic response. To begin this investigation, we determined whether 15d-PMJ_2_ caused apoptosis in HCT116 colon cancer cells. The human HCT116 cell line was selected for this study because it contains mutations in common driver genes (e.g., KRAS and PIK3CA) that serve as targets of contemporary drug development programs (Cancer Genome Atlas Network, 2012; Ahmed et al., 2013). The cells were treated with different concentrations of 15d-PMJ_2_ and then two well-established indicators of apoptosis, caspase 3/7 activity and phosphatidyl serine exposure, were detected. 15d-PMJ_2_ increased caspase 3/7 activity by approximately 200% and 300% in cells treated with 2.5 µM and 5.0 µM of 15d-PMJ_2_, respectively (Figure 1A). 15d-PMJ_2_ also caused a concentration-dependent increase in percentage of early (Annexin V^+^/PI^−^) and late (Annexin V^+^/PI^+^) stage apoptotic cells with 5 µM 15d-PMJ_2_ increasing the total annexin-V positive cell population by more than 60% (Figures 1B, C).

According to our previous melanoma and non-melanoma skin cancer cell studies, PERK-mediated ER stress was the primary molecular pathway of 15d-PMJ_2_-induced apoptosis (Ladin et al., 2022; 2017). During ER stress, PERK undergoes homodimerization and autophosphorylation to assume an activated state. Activated PERK phosphorylates eIF2a, an enzyme that resolves ER stress by inhibiting global translation. In HCT116 cells, ER stress was activated by 15d-PMJ_2_ as demonstrated by increased phosphorylation of both eIF2α and PERK (Figure 1D). To evaluate whether ER stress was necessary for apoptosis, ER stress activation was inhibited by pre-treating the cells with the pharmacological ER stress inhibitors, salubrinal and 4-phenylbutrate (PBA). Salubrinal is a selective inhibitor of the phosphatase, PP1/GADD34 (Boyce et al., 2005). The inhibition of PP1/GADD34 activity prevents the dephosphorylation of phospho-eIF2α, thereby sustaining the translational block and averting ER stress. In contrast, PBA suppresses ER stress by behaving as a chemical chaperone that facilitates protein folding (de Almeida et al., 2007). Blockade of ER stress signaling with salubrinal or PBA suppressed 15d-PMJ_2_-induced caspase 3/7 activity (Figure 1E). Both inhibitors also blocked ER stress-dependent apoptosis induced by the prototype ER stress inducing agent, thapsigargin (Figure 1E). This indicates, ER stress also plays a prominent role in 15d-PMJ_2_-induced apoptosis in HCT116 cells.

### CHOP10 triggers apoptosis induced by 15d-PMJ_2_

**FIGURE 2:**
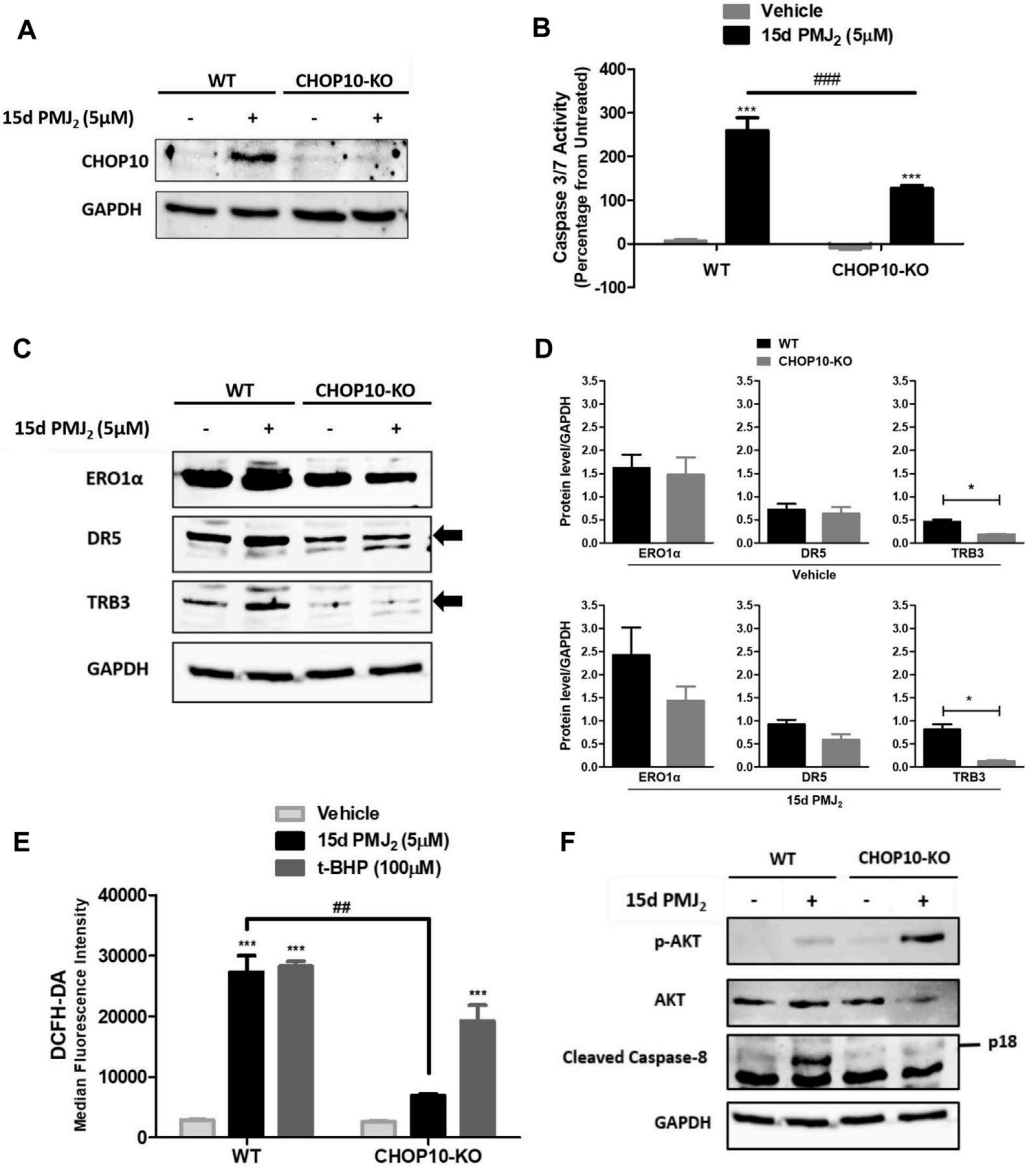
CHOP10 increases the synthesis and activity of TRB3 as well as the activation of apoptosis in 15d-PMJ_2_-treated cells. **(A–F)** Wild-type (WT) and CHOP10 knockout (CHOP10-KO) HCT116 cells were treated with 5 µM 15d-PMJ_2_ or vehicle. **(A)** The cells were treated with 15d-PMJ_2_ or vehicle for 8 h and the expression of CHOP10 (MW: 30 kDa) was detected by conducting western blot analysis. **(B)** Apoptosis was measured by conducting caspase 3/7 activity assays after 16 h of exposure to 15d-PMJ_2_ or vehicle. **(C)** The expression of ERO1α (MW: 54 kDa), DR5 (MW: 50 kDa), and TRB3 (MW: 40 kDa) was measured at 8 h by performing western blot analysis. **(D)** Quantification of ERO1α, DR5, and TRB3 protein expression in vehicle (top)- and 15d-PMJ_2_ (bottom)-treated cells was conducted via densitometric analysis with ImageJ software. **(E)** HCT116-WT and HCT116-CHOP10-KO cells were treated with 5 µM 15d-PMJ_2_, 100 µM tert-butyl hydroperoxide (t-BHP; positive control), or vehicle for 8 h. Oxidative stress was measured by performing flow cytometric analysis using the CM-H_2_DCFDA probe. **(F)** Phosphorylated Akt, total Akt (MW: 60 kDa), cleaved caspase 8 (p18; MW: 18 kDa), and GAPDH were detected by performing western blot analysis. The data were analyzed using two-way ANOVA followed by Bonferroni’s multiple comparison post-test and are presented as the mean ± SEM of three independent experiments performed in triplicate. *p < 0.05, ***p < 0.001 when comparing samples to vehicle-treated cells. ##*p* < 0.01, ###*p* < 0.001 when comparing samples to 15d-PMJ_2_-treated cells.

When ER stress cannot be resolved, the expression of the transcription factor, CHOP10, is upregulated. CHOP10 transactivates proteins that propagate apoptotic signals, including ERO1α, DR5, and TRB3 (Li et al., 2014; Yang et al., 2017; Hu et al., 2019). Similar to our findings in B16F10 melanoma cells, CHOP10 expression in HCT116 cells was at its peak 8 h after exposure to 15d-PMJ_2_. To identify pathways by which CHOP10 regulates cell death induced by 15d-PMJ_2_, CHOP10 expression was disabled by employing the CRISPR/Cas9 system. We validated that the expression of CHOP10 was inhibited by exposing HCT116 CHOP10 knock-out (CHOP10-KO) and wild-type (WT) cells to 15d-PMJ_2_ and detecting CHOP10 expression by western blot analysis (Figure 2A). Consistent with this result, 15d-PMJ_2_-induced caspase 3/7 activity was inhibited by more than 50% in CHOP10-KO compared to WT cells (Figure 2B). Moreover, fewer annexin V positive cells were observed in 15d-PMJ_2_-treated cells that were devoid of CHOP10 than in cells containing CHOP10 (Supplementary Figure S1). These data indicate CHOP10 is needed to elicit a robust apoptotic response to 15d-PMJ_2_.

To identify transcriptional targets of CHOP10 that are pivotal for 15d-PMJ_2_-induced apoptosis, we measured the expression and activity of ERO1α, DR5, and TRB3 in WT and CHOP10-KO cells (Figures 2C–F). ERO1α is an enzyme that promotes protein folding by facilitating disulfide bond formation, but it can also produce cytotoxic oxidative stress levels as a reaction by-product (Marciniak et al., 2004; Chen et al., 2015; Ramming et al., 2015). DR5 is a member of the death receptor family that initiates extrinsic apoptotic signaling by stimulating the cleavage of caspase 8 (MacFarlane et al., 1997; He et al., 2002; Yamaguchi and Wang, 2004). The pseudokinase TRB3, triggers cell death by suppressing the activity of the pro-survival kinase, Akt (Ohoka et al., 2005; Li et al., 2017). In 15d-PMJ_2_ treated cells, the loss of CHOP10 caused a reduction in the expression of ERO1α, DR5, and TRB3 although only the reduction in TRB3 expression was statistically significantly (Figures 2C, D). Next, we examined the effect of CHOP10 on the activity of ERO1α, DR5, and TRB3 in 15d-PMJ_2_-treated cells. Oxidative stress, a by-product of ERO1α activity, was suppressed in cells with disrupted CHOP10 expression compared with intact CHOP10 expression (Figure 2E). Cleaved caspase 8, a mediator of the apoptotic effect of DR5, was upregulated in 15d-PMJ_2_-treated cells with, but not cells without, CHOP10 expression (Figure 2F). In 15d-PMJ_2_-treated cells, CHOP10 containing, but not CHOP10 deficient, cells showed an increase in TRB3 activity, detected as reduced Akt phosphorylation (Figure 2F). These collective data suggest CHOP10, and its down-stream effectors propagate apoptotic signaling induced by 15d-PMJ_2_.

**FIGURE 3:**
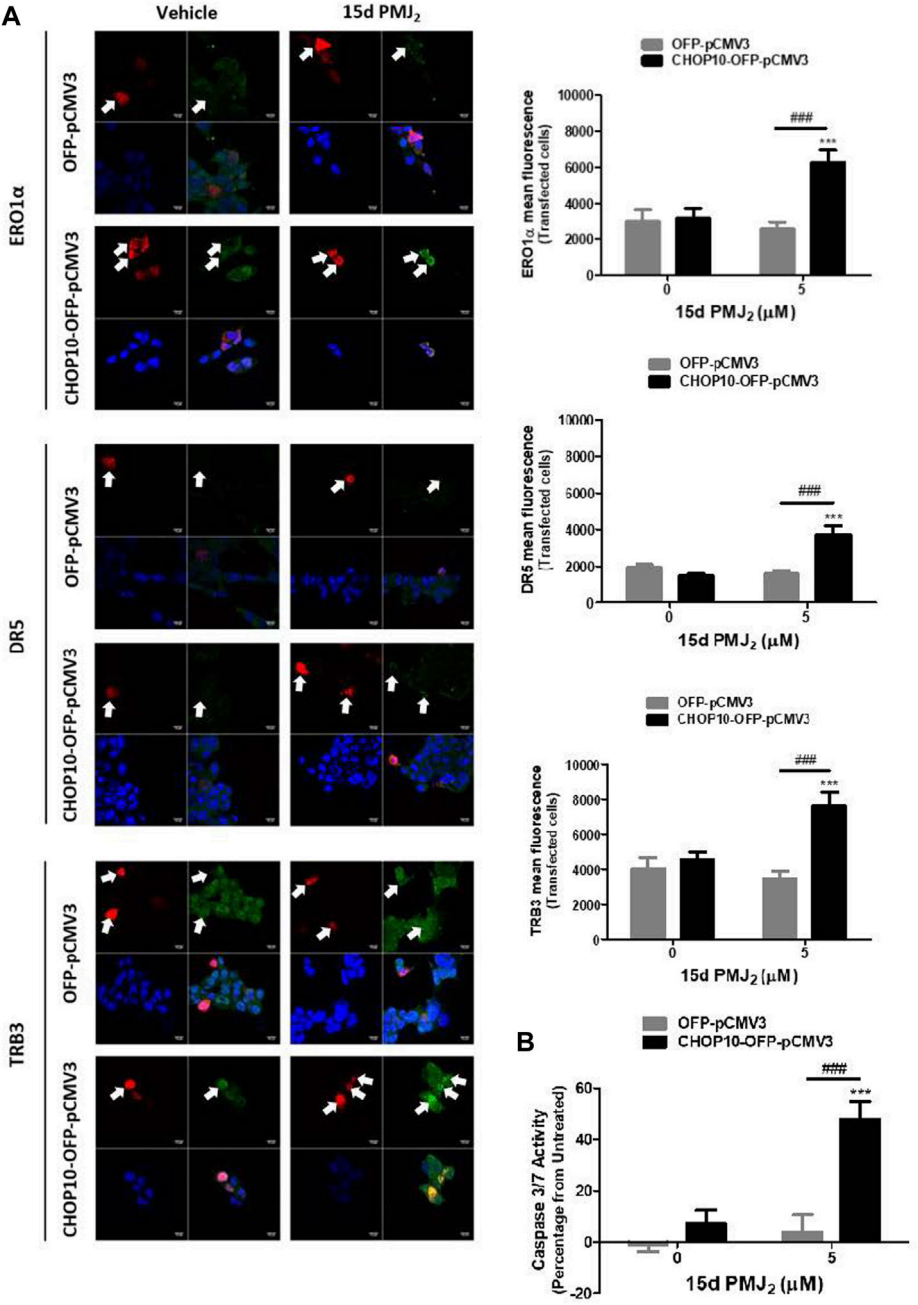
Restoration of CHOP10 expression in CHOP10-KO cells increases the synthesis of ERO1α, DR5, and TRB3 and the activation of apoptosis after exposure to 15d-PMJ_2_. **(A,B)** pCMV3-OFP- or CHOP10-pCMV3-OFP-transfected CHOP10-KO cells were treated with 5 µM 15d-PMJ_2_ or vehicle. **(A)** The expression of ERO1α, DR5, and TRB3 was detected by performing immunofluorescence staining and visualizing the images using confocal microscopy. Green fluorescence represents ERO1α, DR5, or TRB3, red fluorescence represents CHOP10 or empty OFP vector, and blue fluorescence represents the nucleus (DAPI staining). Histograms show quantification of the intensity of ERO1α, DR5, and TRB3. **(B)** Apoptosis measurement was performed using caspase 3/7 activity assays. The data were analyzed using two-way ANOVA followed by Bonferroni’s multiple comparison post-test and are presented as the mean ± SEM of three independent experiments. ***p < 0.001 when comparing samples to vehicle-treated cells. ###*p* < 0.001 when comparing samples to 15d-PMJ_2_-treated cells.

CRISPR/Cas9 is a highly effective method for knocking down gene expression with minimal off-target effects. However, to verify that the CRISPR/Cas9 constructs targeted CHOP10 but not off-target genes, we restored CHOP10 expression by transfecting CHOP10-KO cells with a mammalian expression vector containing CHOP10 cDNA (or the corresponding empty vector). In 15d-PMJ_2_-treated cells that contained CHOP10, the expression of ERO1α, DR5, and TRB3 was significantly increased compared to cells containing the empty vector (Figure 3A). Additionally, the expression of CHOP10 in CHOP10-KO cells increased caspase 3/7 activity after cell exposure to 15d-PMJ_2_ (Figure 3B). This suggests CRISPR/Cas9 specifically targeted CHOP10 and that stimulation of ERO1α, DR5, and TRB3 expression and apoptosis in 15d-PMJ_2_-treated cells was mediated by CHOP10.

### TRB3 stimulates 15d-PMJ_2_ -induced apoptosis downstream of CHOP10

**FIGURE 4:**
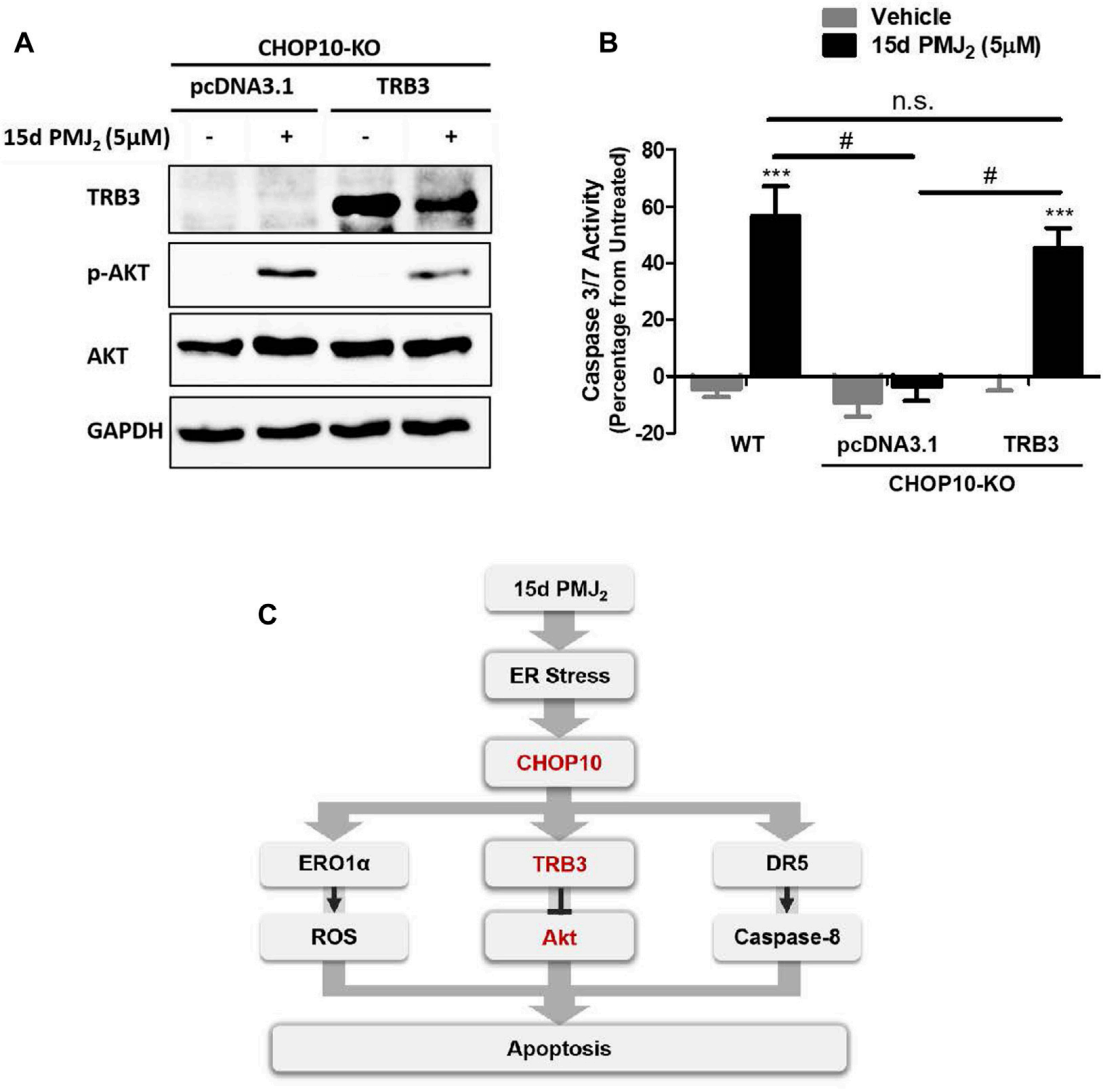
TRB3 inhibits Akt activation and restores apoptosis in 15d-PMJ_2_-treated CHOP10-KO cells. **(A)** CHOP10-KO cells were transfected with pcDNA3.1 or TRB3-pcDNA3.1 and then the cells were treated with 15d-PMJ_2_ or vehicle (0.1% DMSO) for 6 h. Western blot analysis was conducted to analyze TRB3, p-Akt, and Akt protein expression levels. **(B)** CHOP10-KO cells were transfected with pcDNA3.1 or TRB3-pcDNA3.1. The transfected cells and HCT116 WT cells were then treated with 5 µM 15d-PMJ_2_ or vehicle (0.1% DMSO) for 6 h. Apoptosis was detected by conducting caspase 3/7 activity assays. The data were analyzed using two-way ANOVA followed by Bonferroni’s multiple comparison post-test and are shown as means ± SEM of three independent experiments performed in triplicate. ***, *p* < 0.001, when comparing samples to vehicle-treated cells. #, *p* < 0.05, when comparing samples to 15d-PMJ_2_ -treated cells. **(C)** Schematic model illustrating a molecular mechanism of 15d-PMJ_2_-induced apoptosis. 15d-PMJ_2_ activates ER stress by up-regulating CHOP10 expression. CHOP10 significantly increases the expression of pro-apoptotic TRB3. TRB3 regulates apoptosis through the inhibition of Akt.

Our data in Figure 2C show both endogenous and 15d-PMJ_2_-induced expression of TRB3 was significantly decreased in CHOP10 deficient compared to CHOP10 containing cells. This suggests TRB3 may have a critical role in executing CHOP10-mediated apoptosis in response to 15d-PMJ_2_. To examine this signaling pathway in greater detail, TRB3 expression was restored in CHOP10-KO cells by transfecting the cells with a DNA vector containing TRB3 cDNA. In CHOP10-KO cells that were treated with 15d-PMJ_2_, expression of TRB3 suppressed the phosphorylation of Akt (Figure 4A). In line with the reduction in Akt activation, transfection of the TRB3-containing plasmid, but not the empty vector, increased caspase 3/7 activity in 15d-PMJ_2_-treated CHOP10-KO cells with levels comparable to 15d-PMJ_2_-treated WT cells (Figure 4B). This implies that a mechanism of 15d-PMJ_2_-induced apoptosis in HCT116 cells includes the ER stress dependent activation of CHOP10 which causes the upregulation of TRB3, an inhibitor of the anti-apoptotic protein, Akt (Figure 4C).

## Discussion

The current study was undertaken to gain an understanding of molecular pathways by which CHOP10 causes apoptosis in 15d-PMJ_2_-treated tumor cells. We determined 15d-PMJ_2_ activated CHOP10 and its transcriptional product, TRB3, which led to the inhibition of the anti-apoptotic protein, Akt. These findings reveal an important pathway of 15d-PMJ_2_-induced cell death.

It is well documented that the transcription factor, CHOP10, plays a pivotal role in the switch between the ER stress survival and death pathways (Ma et al., 2002; Li et al., 2014; Yang et al., 2017). Our data indicates CHOP10 also regulates the apoptotic properties of 15d-PMJ_2_. In HCT116 colon cancer cells, 15d-PMJ_2_ increased the expression of CHOP10 and the activation of apoptosis in a concentration-dependent manner. However, the apoptotic activity of 15d-PMJ_2_ was suppressed in cells that lacked CHOP10 while restoration of CHOP10 expression reinstated apoptosis. These findings agree with our previous data showing 15d-PMJ_2_ increased CHOP10 expression and cell death in melanoma and NMSC cells (Ladin et al., 2022; 2017). Moreover, AEA and PMD_2_, two pro-apoptotic molecules that are catabolized to 15d-PMJ_2_ in tumor cells that overexpress COX-2, also upregulated CHOP10 expression and apoptosis (Soliman et al., 2016; Elhassanny et al., 2019). Other groups have demonstrated CHOP10 dictates cell fate during ER stress. For example, Lin et al. (2013) showed that capsaicin-induced expression of CHOP10 caused apoptosis in pancreatic cancer cells and that this effect was suppressed by siRNA-mediated downregulation of CHOP10. Also, apoptosis induced by tunicamycin was prevented by blocking the expression of CHOP10 in hepatocellular carcinoma (Lei et al., 2017). Hence, CHOP10 is an important stimulus for initiating apoptosis and agents that trigger its synthesis may be useful components of cancer chemotherapeutic regimens.

Although it is clear that CHOP10 causes apoptosis in response to 15d-PMJ_2_ exposure, the molecular mechanism behind this effect had not been elucidated. Having knowledge about mechanisms of 15d-PMJ_2_-induced cell death can improve the outcome of future clinical studies by allowing the prediction of agent activities including the modes of toxicity and resistance (Editorial, 2010; Lin et al., 2019; Jang et al., 2021). It can also facilitate the discovery of predictive biomarkers, the identification of new drug targets, and the selection of co-administered agents (Editorial, 2010; Davis, 2020). Therefore, as part of our effort to develop 15d-PMJ_2_ as a cancer therapeutic, the current study examined methods of 15d-PMJ_2_ apoptosis. Operating under the knowledge that CHOP10 can transcriptionally activate the pro-apoptotic proteins, ERO1α, DR5, and TRB3, we examined the expression of these molecules. In 15d-PMJ_2_ treated cells, the loss of CHOP10 caused a significant reduction in the expression of TRB3 but not ERO1α or DR5. This indicated the potential relevance of TRB3 in the activity of 15d-PMJ_2_. To examine the effect of TRB3 in greater detail, HCT116 CHOP10-KO cells were transfected with an expression vector containing TRB3 and then treated with 15d-PMJ_2_. Under these conditions, TRB3 suppressed Akt activation and restored apoptosis implying CHOP10/TRB3/Akt inhibition triggered apoptosis. Apoptotic cell death was also mediated by CHOP10/TRB3/Akt inhibition in prostate cancer cells treated with the agent, corosolic acid (Ma et al., 2018). These outcomes are in alignment with data showing TRB3 inhibits Akt through an interaction that hinders Akt phosphorylation and activation (Du et al., 2003). It is also well-known that the blockade of Akt activity stimulates apoptosis by inhibiting anti-apoptotic proteins (Datta et al., 1997). Therefore, it was not surprising that TRB3 expression and the subsequent inactivation of Akt in CHOP10-KO cells restored the apoptotic activity of 15d-PMJ_2_. Since 15d-PMJ_2_ inhibits Akt and activates ER stress, this suggests 15d-PMJ_2_ may enhance the anti-tumor activity of FDA approved cancer therapeutics. For instance, the ER stress inhibitor, salubrinal, promoted differentiation of colon cancer stem cells thereby sensitizing them to the cytotoxic effect of the colon cancer drug, oxaliplatin (Wielenga et al., 2015). The agent, GSK690693, which blocks Akt activity, caused a significant increase in the cytotoxicity of irinotecan compared to cells treated with GSK690693 or irinotecan alone (Pradeepa et al., 2022). As such, our future studies will investigate the cytotoxic effects of combination therapy with 15d-PMJ_2_.

Previous work from our group demonstrated that 15d-PMJ_2_ activated the ER stress response primarily through PERK, but not ATF6 or IRE1 signaling (Ladin et al., 2022). Moreover, ER stress-dependent Ca^2+^ mobilization from the ER to the mitochondria played a crucial role in mPTP-driven apoptosis. Although our collective research has uncovered important mechanisms of 15d-PMJ_2_ activity upstream of CHOP10, we have not identified the specific molecule(s) that interact with 15d-PMJ_2_ to initiate ER stress and apoptosis. Having a detailed knowledge of both the molecular target(s) and activities of 15d-PMJ_2_ will permit us to apply this information towards the development of optimized therapeutic regimens and as such, our efforts are focused on these topics.

The current study investigated mechanisms of 15d-PMJ_2_-induced apoptosis as part of our assessment of its activity and chemotherapeutic potential. We determined 15d-PMJ_2_ caused tumor cell apoptosis through the activation of ER stress and CHOP10. The activity of CHOP10 stimulated TRB3 expression which led to the inhibition of the pro-survival kinase, Akt. This identifies CHOP10/TRB3/Akt inhibition as vital for the effects of 15d-PMJ_2,_ and it reveals a molecular mechanism underpinning the activity of 15d-PMJ_2_. By stimulating this signaling pathway, 15d-PMJ_2_ may provide an effective strategy for killing cancer cells.

### Limitations of the study

Although we have uncovered signaling pathways involved in 15dPMJ_2_-induced apoptosis, there are two notable shortcomings in this study that are being addressed in our current and upcoming work. First, 15d-PMJ_2_-induced apoptotic signaling was examined in wild-type HCT116 and CHOP10-KO HCT116 human cell lines, but not in additional cancer cell types. To obtain a clear picture of the signaling pathways that lead to death in other cell lines, a complete loss of CHOP10 function is essential. Pharmacological inhibitors that selectively target CHOP10 are not readily available, but the current study and other reports (Guo et al., 2021) demonstrate CRISPR/Cas9 gene editing allows the identification of signaling pathways that are downstream of CHOP10. Using this strategy, we will knockout the CHOP10 gene in different cell lines to investigate pathways utilized by 15d-PMJ_2_ to elicit cell death. Second, the *in vitro* activity of 15d-PMJ_2_ was investigated without examining its action an animal tumor model. Previous work from our group demonstrated 15d-PMJ_2_ inhibited the growth of B16F10 melanoma in C57BL/6 mice (Ladin et al., 2017). We will examine the activity of 15d-PMJ_2_ by implanting cell lines with genetically ablated CHOP10 into the subcutaneous flank of immunocompromised mice. These studies will provide a comprehensive understanding of CHOP10-mediated signaling and the therapeutic potential of 15d-PMJ_2_.

## Data Availability

The original contributions presented in the study are included in the article/Supplementary Material, further inquiries can be directed to the corresponding author.

## Abbreviations

15d-PGJ_2_, 15-deoxy-Δ^12,14^ -prostaglandin J_2_; 15d-PMJ_2_, 15-deoxy-Δ^12,14^ -prostamide J_2_; 4-PBA, 4-phenylbutrate; AEA, Arachidonoyl-ethanolamide (also known as anandamide); ANOVA, Analysis of Variance; ATF6, Activated transcription factor 6; CHOP10, C/EBP homologous protein 10; CM-H 2, DCFDA Chloromethyl-2′, 7′ dichlorodihydrofluorescein diacetate; DMSO, Dimethyl sulfoxide; DR5, Death receptor 5; eIF2α, Eukaryotic initiation factor −2 alpha; ER, Endoplasmic reticulum; ERO1α, ER oxidoreductase 1 alpha; FBS, Fetal bovine serum; GAPDH, Glyceraldehyde 3-phosphate dehydrogenase; IRE1, Inositol requiring kinase-1; NMSC, Non-melanoma skin cancer; PBS, Phosphate buffered saline; PERK, Double-stranded RNA-activated protein kinase (PKR)-like endoplasmic reticulum kinase; PG, Prostaglandin; PGD2-EA, Prostaglandin D2-ethanolamide; PM, Prostamides; PMD2, Prostamide D2; P-PERK, Phosphorylated PERK; ROS, Reactive oxygen species; SEM, Standard error of the mean; TLB, Triton lysis buffer; TNF, Tumor necrosis factor; TRAIL, TNF-related apoptosis-inducing ligand; TRB3, Tribbles-related protein 3.
