# Supplementary figures and images for: Apoptosis mechanisms induced by 15d-PMJ2 in HCT116 colon cancer cells: insights into CHOP10/TRB3/Akt signaling
Source: Front Pharmacol. 2023 Nov 2;14:1283677. doi: 10.3389/fphar.2023.1283677 (PMC10652392; doi:10.3389/fphar.2023.1283677)

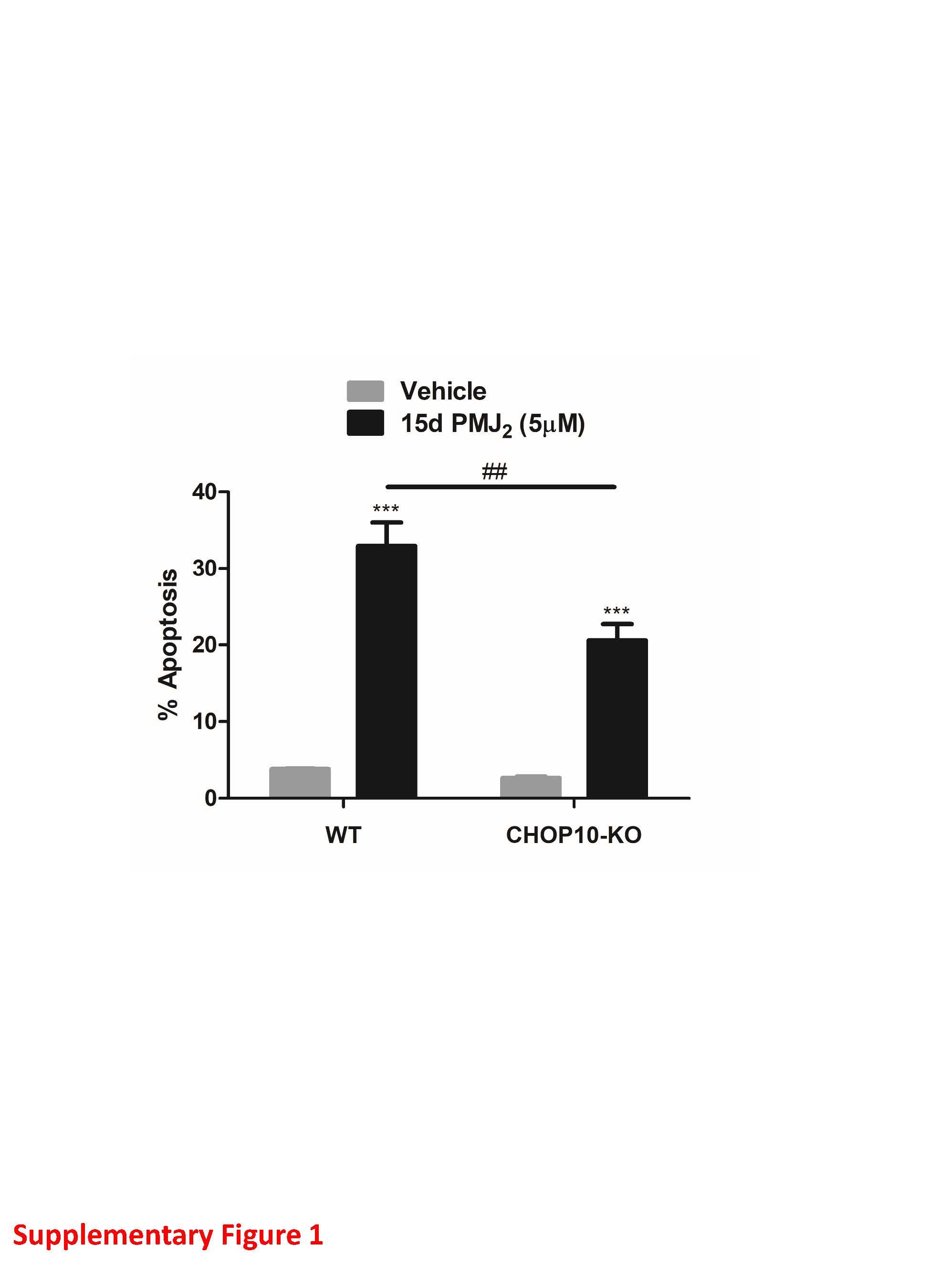

Supplement: Supplementary file 1 [file Image1.JPEG]
