# Supplementary material for: Apoptosis mechanisms induced by 15d-PMJ2 in HCT116 colon cancer cells: insights into CHOP10/TRB3/Akt signaling
Source: Front Pharmacol. 2023 Nov 2;14:1283677. doi: 10.3389/fphar.2023.1283677 (PMC10652392; doi:10.3389/fphar.2023.1283677)
Supplement: Supplementary file 2 [file DataSheet1.PDF]

Supplementary Figure 1. CHOP10 is essential for 15d-PMJ$_2$-induced apoptosis. HCT116-WT and HCT116 CHOP10-KO cells were treated with 5 µM 15d-PMJ$_2$ or vehicle (medium containing ≤0.1% DMSO). Apoptosis was measured at 12 hours by conducting flow cytometric analysis to detect Annexin-V+ cells (% apoptosis compared to untreated cells). The data were analyzed using two-way ANOVA followed by Bonferroni's multiple comparison post-test and are shown as means ± SEM of three independent experiments performed in triplicate. ***, $p<0.001$ when comparing samples to vehicle-treated cells. ##, $p<0.01$; when comparing WT to CHOP10-KO cells.
